# Pathophysiological-Based Nutritional Interventions in Cirrhotic Patients with Sarcopenic Obesity: A State-of-the-Art Narrative Review

**DOI:** 10.3390/nu16030427

**Published:** 2024-01-31

**Authors:** Ernestina Santangeli, Chiara Abbati, Rusi Chen, Alma Di Carlo, Simona Leoni, Fabio Piscaglia, Silvia Ferri

**Affiliations:** 1 Department of Medical and Surgical Sciences, University of Bologna, 40126 Bologna, Italy; ernestina.santangeli@studio.unibo.it (E.S.); chiara.abbati@studio.unibo.it (C.A.); rusi.chen@studio.unibo.it (R.C.); fabio.piscaglia@unibo.it (F.P.); 2 Division of Internal Medicine, Hepatobiliary and Immunoallergologic Diseases, IRCCS Azienda Ospedaliero-Universitaria di Bologna, 40138 Bologna, Italy; alma.dicarlo1@gmail.com (A.D.C.); simona.leoni@aosp.bo.it (S.L.)

**Keywords:** cirrhosis, sarcopenic obesity, inflammation, hyperammonemia, insulin-resistance, nutrition, late evening snack, nutraceuticals, branched-chain amino acids, micronutrients

## Abstract

In recent decades, following the spread of obesity, metabolic dysfunction has come to represent the leading cause of liver disease. The classical clinical presentation of the cirrhotic patient has, therefore, greatly changed, with a dramatic increase in subjects who appear overweight or obese. Due to an obesogenic lifestyle (lack of physical activity and overall malnutrition, with an excess of caloric intake together with a deficit of proteins and micronutrients), these patients frequently develop a complex clinical condition defined as sarcopenic obesity (SO). The interplay between cirrhosis and SO lies in the sharing of multiple pathogenetic mechanisms, including malnutrition/malabsorption, chronic inflammation, hyperammonemia and insulin resistance. The presence of SO worsens the outcome of cirrhotic patients, affecting overall morbidity and mortality. International nutrition and liver diseases societies strongly agree on recommending the use of food as an integral part of the healing process in the comprehensive management of these patients, including a reduction in caloric intake, protein and micronutrient supplementation and sodium restriction. Based on the pathophysiological paths shared by cirrhosis and SO, this narrative review aims to highlight the nutritional interventions currently advocated by international guidelines, as well as to provide hints on the possible role of micronutrients and nutraceuticals in the treatment of this multifaceted clinical condition.

## 1. Introduction

Physiologically, the liver plays a central role in nutritional metabolism, including glucose homeostasis, protein synthesis and drug/toxin metabolism. With the establishment and progression of chronic liver disease, a clinical condition, characterized by the presence of malnutrition, sarcopenia and overall frailty, develops in more than 50% of cirrhotic patients, significantly conditioning overall morbidity and mortality due to a reduced quality of life and hepatic decompensation [1].

In the last decades, following the spread of non-communicable chronic diseases in the general population, together with the development of pharmacological therapies that have radically changed the prognosis of viral hepatitis, metabolic dysfunction has come to represent the leading cause of liver disease (formerly non-alcoholic fatty liver disease—NAFLD, more recently redefined as metabolic dysfunction-associated steatotic liver disease—MASLD) [1]. The classical clinical presentation of the cirrhotic patient has therefore greatly changed in recent years, with a dramatic increase in the percentage of subjects who no longer appear undernourished and underweight but rather normal weight or even overweight or obese. However, a weight within the limits or even above the norm does not necessarily reflect an adequate nutritional status. Indeed, these patients are often characterized by a state of overall poor nutrition, with caloric excess and protein and micronutrient deficiencies, representing the nutritional basis of the complex clinical condition called sarcopenic obesity (SO), whose presence worsens the outcome of the condition of cirrhosis, already burdened by a challenging prognosis on its own.

Despite the lack of SO-specific nutritional intervention trials in patients with liver cirrhosis, multiple recommendations from nutrition and liver diseases societies are available to guide the use of food as an adjuvant therapy in the comprehensive management of these patients, including reduction in caloric intake, protein supplementation, sodium restriction and micronutrient supplementation [1,2].

Based on the pathophysiological background of SO associated with cirrhosis, this narrative review aims to highlight the nutritional interventions currently advocated by international guidelines and to provide hints on the possible role of micronutrients and nutraceuticals in the treatment of this multifaceted clinical condition.

## 2. Materials and Methods

We searched full-text English-language publications in MEDLINE, Ovid, the Cochrane Library and Pubmed, focusing on the pathophysiological basis and nutritional interventions in cirrhosis with sarcopenic obesity from inception to August 2023. The initial keywords were: “nutrition” OR “frailty” OR “obesity” OR “sarcopenia” OR “sarcopenic obesity” AND “liver cirrhosis” OR “chronic liver disease”. Further, more specific keywords were also used: “cholestatis” OR “alcohol-related liver disease” OR “dysmetabolic liver disease” OR “metabolic syndrome” OR “hepatic encephalopathy” OR “nutrient deficit” OR “nutritional supplementations”. The references for these papers were reviewed as well to find additional manuscripts for consideration in this narrative review.

## 3. Cirrhosis with SO: Clinical Aspects

Given the liver’s central role in synthesizing, storing, and metabolizing nutrients, it is not surprising how liver disease can affect all these processes. In carbohydrate metabolism, the liver represents the central player in anabolic and synthesizing pathways, including glucose and glycogen synthesis, as well as in the catabolic processes of glycolysis and glycogenolysis. In amino acid and lipid metabolism, the liver has a predominant anabolic role in the synthesis of many serum proteins (e.g., albumin, coagulation factors, anticoagulant factors and inflammation proteins), triglycerides, cholesterol and lipoproteins. Additionally, the liver produces and excretes bile salts, which are essential for the intestinal absorption of dietary fats and fat-soluble vitamins. Lastly, the liver is the principal site for detoxification of substances and metabolites coming from the systemic and portal circulatory streams, including ammonia. Thus, when liver function is severely impaired due to acute or chronic diseases, all these pathways are somehow affected [3].

Currently, liver cirrhosis is the 11th leading cause of death and the 15th leading cause of morbidity, accounting for 2.4% of deaths and nearly 41.4 million of disability-adjusted life years worldwide in 2017 [4]. Liver cirrhosis represents the common end stage of any condition causing chronic liver injury and fibrosis, thus resulting in liver dysfunction and portal hypertension [5]. It usually remains asymptomatic in its early phases until the occurrence of one of its complications, such as ascites, spontaneous bacterial peritonitis, hepatic encephalopathy, variceal bleeding, coagulopathy and hepatocellular carcinoma [5,6]. In Western countries, metabolic dysfunction-associated steatotic liver disease (MASLD), excessive alcohol consumption and chronic HCV infection are the most common causes of liver cirrhosis, whereas chronic HBV infection represents the primary etiology in Asia. Other causes are inherited diseases, such as hemochromatosis and Wilson’s disease, and immune-mediated liver diseases (primary biliary cholangitis—PBC, primary sclerosing cholangitis—PSC, and autoimmune hepatitis—AIH) [7,8]. Due to the irreversibility of the cirrhotic condition and the current lack of an effective and validated antifibrotic medical treatment, the therapy for advanced liver disease relies on the removal of its underlying causes, prevention and management of acute and chronic complications and, when indicated, liver transplantation [5].

Sarcopenia is a generalized and progressive skeletal muscle disorder, characterized by low muscle quality and quantity. A first consensus held in 2010 by the European Working Group on Sarcopenia in Older People (EWGSOP1) [9] focused mainly on the loss of muscle mass, whereas the more modern approach of EWGSOP2 (2019) [10] defines sarcopenia as a combination of decreased muscle mass and function, with the latter aspect being predominant in predicting overall adverse outcomes [9,10,11]. Sarcopenia has long been considered mainly age-related, but the new functional definition has also expanded its prevalence to younger people under particular conditions, suggesting that it is a more complex process [10,12]. Sarcopenia can be categorized as primary (age-related, without other specific causal factors) and secondary to disease (inflammatory conditions, malignancy or organ failure), inactivity or poor nutrition [9], and it has been associated with several adverse health-related outcomes. In particular, in liver diseases, the prevalence of sarcopenia increases with liver function impairment, as expressed by the Child–Pugh (CP) score (10% of patients in CP-A, 34% in CP-B and 54% in CP-C), with a rate of annual loss of muscle mass that in cirrhotic patients doubles that of the average elderly Japanese [13,14,15]. Sarcopenia is influenced by the etiology of liver disease, being more frequent (up to 80% of cases) and more rapid to develop in alcohol-related cirrhosis due to direct ethanol toxicity on muscular tissue [16]. In general, sarcopenia is associated with a 1.72 times higher mortality in cirrhotic patients, independent of validated liver-related risk factors, such as the MELD score, the Child–Pugh score and age [17,18], due to prolonged hospitalizations and higher frequency of infections and liver-related complications [17,19,20,21]. Sarcopenia affects the outcome before and after liver transplantation, independent of liver function scores: mortality rates among individuals on the waiting list and post liver transplantation are significantly higher in sarcopenic compared to non-sarcopenic patients, and sarcopenic patients with a low MELD score experience outcomes similar to non-sarcopenic subjects with worse liver function [14,22]. In particular, the recovery period after transplantation is prolonged in patients with sarcopenia due to a longer period of rehabilitation and a higher risk of bacterial infections caused by malnutrition and impaired immunity, though a direct effect of sarcopenia on overall survival after liver transplantation is still debated [23,24]. Due to the fact that sarcopenia is a predictor of mortality and complications after liver transplantation, a new score (MELD-sarcopenia) has been proposed to favor a better organ allocation, in particular in sarcopenic patients with lower MELD scores [14,17,25].

Currently, the biometric index used to define patients’ weight status is BMI (body mass index), which is calculated as the ratio of a person’s weight to the square of his/her height. According to the European guidelines for adults (aged over 18 years), obesity is defined by a BMI > 30 kg/m^2^ and overweight by a BMI between 25 and 29.9 kg/m^2^. Obesity is considered a chronic metabolic disease and it has been recognized as one of the leading causes of disability (including diabetes, hypertension and dyslipidemia) and death [26]. The risk of death for all causes (mainly cardiovascular diseases, cancers and type 2 diabetes) increases with higher BMIs, determining a reduction in life expectancy by 6.5–13.7 years, compared to the control population, for subjects with BMI > 40 [27,28]. Nowadays, together with the obesity and overweight epidemic spread [29], the prevalence of dysmetabolic liver disease (MASLD—metabolic dysfunction-associated steatotic liver disease, MASH—metabolic dysfunction-associated steato hepatitis, dysmetabolic cirrhosis and HCC—hepatocarcinoma) is growing, now ranking as the leading cause of liver diseases in the Western world. However, the presence of excessive weight is also often detected in patients with liver disease of different etiologies, further complicating the clinical picture [1]. The estimated global prevalence of MASLD in 2019 was approximately 37%, with a steady increase over time (0.7% annual increase from 1990) [30]. The obese population displays a higher risk of developing chronic liver diseases compared to the general population: the large prospective study ‘Million Women Study’ conduced in the UK demonstrated that the risk of liver cirrhosis increases by about 28% for each 5 unit increase in BMI [31], not only when the underlying liver disease is MASLD, but also when there is a viral or alcohol-related etiology [32,33,34]. More advanced stages of liver disease are most commonly found when all components of the metabolic syndrome (visceral obesity, arterial hypertension, dyslipidemia, type 2 diabetes mellitus) are present or with higher BMIs [35]. In particular, obesity seems to be also associated with an increased risk of cirrhosis decompensation, including both spontaneous occurrences and complications from therapeutic interventions [36,37,38], and primary liver cancer development [39].

Sarcopenic obesity (SO) is a clinical and functional condition, precisely defined in the last decade, where obesity and sarcopenia coexist [40]. This condition, characterized by loss of muscle mass in favor of adipose tissue, is detected in cirrhotic patients more and more frequently due to the widespread prevalence of metabolic syndrome, reaching up to 20% of cases in some series [1,36,41]. Obesity-associated sarcopenia is not due to *under*-nutrition but rather to *mal*-nutrition, with a typical high-energy but poor-quality dietary intake. The sedentary lifestyle often led by these patients further contributes to loss of muscle mass and function due to inactivity. Since its recent definition, the selective analysis of the subgroup of cirrhotic patients with SO has already provided some interesting data, suggesting that the presence of sarcopenic obesity may worsen the prognosis of patients with liver cirrhosis, with an additive effect compared to the two conditions considered separately [1]. In a Japanese cohort of 161 cirrhotic outpatients of different etiologies followed up for roughly 3 years, 67% of subjects with SO died, compared to 48% and 36% of those with sarcopenia and visceral obesity alone, with a significance that was evident in the Child-A subgroup but diminished in more advanced stages of disease [41]. In a recent study analyzing an American cohort of 326 cirrhotic patients on the liver transplantation waiting list, the coexistence of sarcopenia and obesity accounted for an independent mortality hazard ratio of 2.64, more than double that of the two conditions occurring separately [36].

Myosteatosis, a pathological fatty infiltration of skeletal muscle, develops when adipocytes’ maximum capacity to store fat is exceeded (as in excessive weight gain or when subcutaneous tissues develop a decreased storage ability) but also in physiological aging (possibly due to age-related differentiation of muscle stem cells into adipocytes) and in chronic inflammation or dysmetabolic conditions characterized by insulin-resistance [42,43,44]. Excessive fat accumulation in muscles may impact muscle fiber orientation, determining tissutal inflammation and atrophy. Indeed, myosteatosis has been associated with reduced muscle strength and physical performance, lastly leading to an overt sarcopenic condition with increased disability [45,46]. In cirrhotic patients, myosteatosis is also favored by an excessive ammonia concentration into myocells., This elevated ammonia level, via mitochondrial dysfunction, favors fat accumulation [47]. The detrimental effects of myosteatosis on cirrhotic patients’ outcomes, regardless of weight or adiposity, have been proven by several studies both in terms of morbidity, especially hepatic encephalopathy at all degrees, and mortality [48,49,50]. Due to its impact on mortality, the duration of intensive care unit stay and short-term complication rates in patients receiving deceased donor orthotopic liver transplant, the presence of myosteatosis has been proposed to be taken into account to optimize donor/recipient combinations and organ allocation [51].

## 4. Cirrhosis with SO: Pathophysiological Aspects

In a healthy condition, the liver, muscles and adipose tissue act together to sustain metabolic balance, so it is not surprising that, even when pathology develops, these three organs share common metabolic pathways, ultimately establishing a self-maintaining vicious circle, characterized by the loss of mass and function in “noble tissues” such as (but not only) the skeletal muscle.

**Figure 1:**
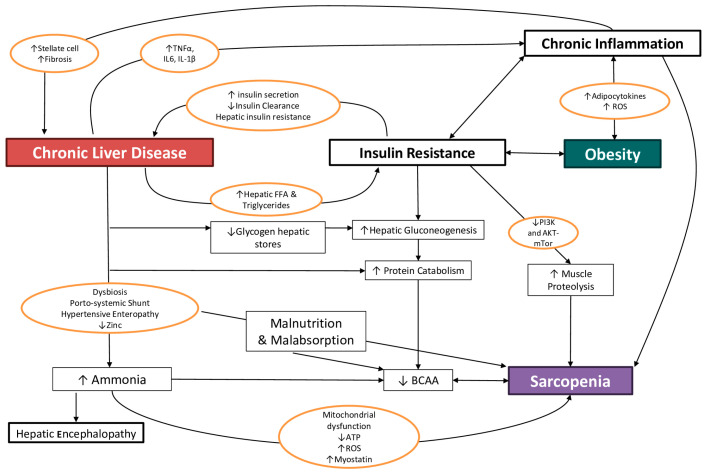
The main interplays linking chronic liver disease, obesity and sarcopenia. ROS: reactive oxygen species, BCAA: branched-chain amino acids, FFA: free fatty acids, TNF: tumor necrosis factor, IL: interleukin, PI3K: phosphatidyl-inositol 3-kinase, AKT (or PKB): a serine/threonine-specific protein kinases, mTOR: mammalian target of rapamycin, ATP: adenosine triphosphate.

The main mechanisms contributing to this condition, with different weights depending on the grade and stage of hepatic dysfunction, are illustrated below (Figure 1):

In the context of this complex interplay, this review will focus on the mechanisms through which the efficacy of a nutritional intervention has been ascertained or assumed.

### 4.1. Malnutrition and Malabsorption

A reduced daily food intake is frequently reported in cirrhotic patients, especially during decompensation: ascites, compressing the stomach, can cause early satiety and less appetite, whereas hepatic encephalopathy may determine a difficulty in carrying out daily activities, including preparing meals or consuming food [52,53]. In addition, to avoid water retention and the appearance of edema and ascites, the cirrhotic patient is frequently instructed to maintain a low-salt diet (less than 2 g/day), but foods with less salt are less palatable, with an impact on the reduction in food intake, further promoting malnutrition and sarcopenia [54]. Even in the compensated stages of the disease, dysgeusia, possibly due to the combined deficiency of zinc and vitamin A, involved in maintaining taste bud activity, is frequently reported by patients with cirrhosis, with the consequence of a poor and monotonous diet, leading to a higher risk of nutritional deficiencies [54]. As emerging from case-control surveys, cirrhotic patients report less healthy eating patterns compared to the general population, with lower consumption of legumes, proteins, vegetable fats and unsweetened beverages and higher consumption of ultra-processed foods. This behavior is even more evident in presence of overweight or obesity [55]. In cirrhotic patients with obesity, the chance of developing sarcopenia has been related to a low consumption of dairy products and vegetables and a higher consumption of alcohol and sweets [56]. Ultra-processed products display worse nutritional qualities compared to natural foods (they are high in energy, salt, free sugars and saturated fats, while being low in fiber and vitamins) but come with a cheaper price and greater palatability due to industrial processes and the use of food additives [57,58]. Extensive consumption of ultra-processed foods has been associated not only with visceral fat accumulation but also with direct liver damage and intestinal microbiota alterations, which plays a possible role in sustaining the proinflammatory milieu that favors the progression of liver disease to the end-stage condition [58].

In cirrhosis, nutrient malabsorption is due to different mechanisms, and it is present in at least 70% of patients with non-alcohol-related disease, in 50% of patients with alcohol-related cirrhosis, and in all those with severe obstructive bile duct disease [59]. Patients with dysmetabolic cirrhosis and obesity display a condition of chronic inflammation with production of cytokines (at first IFN-gamma and then TNF-alpha), which cause alterations in intestinal tight junctions and in gut microbiota, thus compromising the function and integrity of the intestinal barrier. The same mechanism happens in patients with alcohol-related cirrhosis, through direct damage from alcohol itself or from its metabolites (at first acetaldehyde and then ethyl esters) to the intestinal mucosa and the gut microbiome. The resulting dysbiosis and small intestine bacterial overgrowth (SIBO), frequently detected in cirrhotic patients, contribute to the malabsorption of macro- and micronutrients [60,61]. Furthermore, the cirrhotic patient with portal hypertension may develop a condition known as hypertensive enteropathy, which causes edema of the intestinal wall and dilation of intercellular spaces, with reduced absorption of substances, altered intestinal wall permeability and malabsorption [62,63]. Patients with excessive alcohol consumption may also develop chronic pancreatitis, resulting in a reduction in pancreatic enzymes production, leading to fat malabsorption. In fact, pancreatic enzymes are essential in the cleavage of triglycerides into monoglycerides and long-chain fatty acids, which combine with bile acids and phospholipids to form micelles, facilitating their passage through enterocytes and allowing their absorption [64]. In addition, hepatic cholestatic diseases, such as primary sclerosing cholangitis (PSC) and primary biliary cholangitis (PBC), are characterized by enhanced fat and fat-soluble vitamin (vitamin D, A, K, E) malabsorption, secondary to a decreased production and excretion of bile acids into the intestinal lumen [59,65]. Finally, the presence of portosystemic shunts, as a consequence of portal hypertension, causes nutrients to bypass the liver without being processed [66].

### 4.2. Proinflammatory State and Hypermetabolism

Cirrhosis is a proinflammatory condition characterized by high serum levels of cytokines such as TNF-α, IL-6 and IL-1β, together with a decrease in anti-inflammatory molecules [67]. In compensated cirrhosis, DAMPs (damage-associated molecular patterns), produced by necrotic hepatocytes, are mainly responsible for sterile inflammation [68], whereas, in de-compensated cirrhosis, the systemic inflammation is related to portal hypertension, gut dysbiosis and bacterial translocation from the intestinal lumen into the blood, being preferentially sustained by PAMPs (pathogen-associated molecular patterns), with an overall imbalance in favor of proinflammatory cytokines [69]. This mechanism is amplified by the presence of obesity, as excessive visceral fat increases proinflammatory adipocytokines’ levels and ROS (reactive oxygen species) production, leading to an augmented systemic oxidative stress [70]. Chronic inflammation, promoted by the condition of obesity or insulin resistance, can up-regulate the synthesis of connective tissue growth factor by hepatic stellate cells, thus contributing to liver fibrosis [71]. On the other hand, high circulating levels of pro-inflammatory cytokines (particularly IL-6 and TNF-α) are responsible for inappropriate muscle autophagy through the activation of ubiquitin-proteasome pathways in muscle cells, leading to skeletal muscle wasting [11,72]. In particular, in patients with sarcopenic obesity and alcohol use-related disorders, muscular strength deficit has been related to higher serum proinflammatory cytokine levels [73]. Interestingly, in recent years, a number of studies have focused on the inflammatory potential of diet, leading to the development of a population-based dietary inflammatory index (DII) [74]. Higher DII scores have been associated with sarcopenia, especially in overweight/obese subjects [75]. The self-maintaining systemic inflammation has been linked to hypermetabolism, a condition so frequently detected in the advanced stages of liver disease that it is considered as an extrahepatic manifestation of liver failure [76,77]. In end-stage liver disease, hypermetabolism persists due to depleted hepatic glycogen stores. Hypermetabolism is also sustained by increased liver gluconeogenesis, which uses amino acids derived from protein catabolism, a process that strictly links cirrhosis to muscular waste and sarcopenia [11,78].

### 4.3. Hyperammonemia

Ammonia, a product derived through protein catabolism and intestinal bacterial metabolism, is physiologically transferred via portal circulation to the liver, where it is converted to urea via the hepatic urea cycle, and finally excreted by the kidneys. In liver disease, the urea cycle efficacy is reduced, mainly because of enzymatic dysfunction and cofactor deficiency (such as zinc), with a consequent increase in the serum ammonia levels. A further increase in ammonia serum levels in cirrhosis is due to porto-systemic shunts, through which gut ammonia flow directly into the systemic circulation, bypassing liver detoxification. The serum ammonia is then picked up by skeletal muscle, where it is detoxified to glutamate/glutamine by the citric acid cycle. The augmented amounts of ammonia entering the cycle display various effects, favoring sarcopenia: mitochondrial dysfunction, with increased oxidative stress, impaired energy production and lipid oxidation, ending in fat accumulation; depletion of substrates (BCAAs) for muscle protein synthesis [11,47,79]; and transcriptional upregulation of myostatin, a TGF-β superfamily member that reduces muscle protein synthesis, finally leading to muscle cells destruction [80,81]. Indeed, increased myostatin levels have been related to muscle wasting, reduced functional liver reserve and overall survival in cirrhotic patients [82,83]. In turn, sarcopenic patients, due to the loss of muscle mass, have a reduced ammonia detoxification capacity, leading to an increase in ammonia levels in both serum and the brain and a higher risk of encephalopathy [80,84] due to several mechanisms: an increase in the passage of aromatic amino acids (tryptophan, phenylalanine and tyrosine) across the blood–brain barrier, leading to an imbalance that favors inhibitory neurotransmitters [85]; direct activation of the GABAergic system and ammonia accumulation in astrocytes, determining intracellular edema [86].

### 4.4. Insulin-Resistance

Insulin is a hormone with anabolic properties, produced by Langerhans β-cells islets within the pancreas, whose main function is to reduce blood glucose levels by favoring its uptake and use by peripheral tissues [87]. When tissues exhibit poor sensitivity to insulin (insulin-resistance), glucose cannot enter the cells, remaining in the bloodstream. In the first phases of this process, an increase in insulin secretion (hyperinsulinemia) compensates for the peripheral resistance, but when insulin response is no longer adequate to the demands, a hyperglycemic state is established, which may progressively evolve to type 2 diabetes mellitus [87].

In overweight and obese subjects, all tissues are chronically exposed to high levels of metabolic substrates. These are first physiologically stored as triglycerides and glycogen in adipose tissue, liver and muscles, but when the storing capacities of the specialized tissues are exceeded, the chronic exposure to excessive levels of nutrients determines cellular dysfunctions, including increased intracellular and ectopic lipid deposits (lipotoxicity), abnormal protein modification (such as glycation) and increased mitochondrial stress. Finally, the chronic tissutal exposure to excessive nutrients and insulin levels leads to persistent inflammation and alterations in insulin signaling pathways that prevent further glucose influx into already overloaded cells, thus configuring a condition of cellular insulin resistance [88].

Insulin-resistance and hyperinsulinemia are more frequent in cirrhotic patients compared to the healthy population [89]. Hyperinsulinemia is due to both a higher insulin secretion by the pancreas and reduced hepatic clearance [90]: physiologically, the liver degrades 60% of the insulin secreted by the pancreas during the first passage, but in advanced chronic liver diseases, this process is compromised by porto-systemic shunts and liver failure, resulting in a reduced insulin clearance of up to 40% [91]. Moreover, MASLD seems to directly cause hepatic insulin resistance, possibly through chronic inflammation, even in lean subjects with normal glucose serum levels and blood sugar curves [89]. On the other hand, a state of insulin resistance can promote liver disease by increasing hepatic free fatty acids uptake and triglyceride synthesis, resulting in hepatic fat accumulation. As fat accumulation induces mitochondrial fatty acids oxidation, with the production of free oxygen radicals, insulin resistance ultimately contributes to the development of MASH [71].

In healthy muscles, the contemporary presence of high levels of insulin and essential amino acids determines an anabolic stimulus to protein synthesis through the activation of PI3K (phosphatidyl-inositol 3-kinase) and AKT-mTOR pathways (AKT or PKB: a serine/threonine-specific protein kinases, mTOR: mammalian target of rapamycin). Instead, in a situation of insulin resistance, these pathways are down-regulated, with a reduction in protein synthesis, together with the activation of the apoptotic and ubiquitin-proteasome systems, finally leading to accelerated muscle proteolysis and loss of lean body mass [11,92,93].

### 4.5. Micronutrient Deficiencies

#### 4.5.1. Vitamins

*Fat-soluble vitamin* (D, E, and K) deficiencies are frequently detected in chronic liver disease due to reduced oral intake, malabsorption, impaired liver synthesis of carrier and transfer proteins, cholestasis (with deficiency of bile salts, which are required for solubilization and micelle formation), bacterial overgrowth [94,95] and, in cases of coexisting obesity, a further reduction in circulating vitamin levels may occur due to sequestration in adipose tissue deposits.

Vitamin D is a hormone with pleiotropic effects beyond its role in bone homeostasis; in liver in particular, active vitamin D modulates the immune system, favoring an intrahepatic anti-inflammatory and anti-fibrogenic milieu [96]. Vitamin D deficiency (VDD, defined as 25(OH)D levels < 50 nmol/L or <20 ng/mL) is highly frequent (up to 90% of cases in some series) in patients with chronic liver disease, where it correlates with the degree of hepatic dysfunction [97,98,99,100] due to a combination of different mechanisms, such as reduced production of vitamin D-binding proteins and defective formation of the active metabolite of vitamin D, a sedentary lifestyle leading to reduced exposure to sunlight, consumption of foods low in vitamin content and sarcopenia with proportional increase in fat mass [101]. VDD emerged as a clear predictor of mortality in patients with liver disease, showing an association with increased portal hypertension (documented via HVPG), a higher frequency of infectious complications and an overall higher risk of death [98,102,103,104,105,106]. A strong association between VDD and obesity has been demonstrated, likely due to enhanced sequestration in body fat compartments of the lipophilic vitamin D [107]. The active form of vitamin D exerts a direct regulatory role in skeletal muscle function, where it participates in myogenesis, cell proliferation, differentiation, regulation of protein synthesis and mitochondrial metabolism through activation of various cellular signaling cascades, including the mitogen-activated protein kinase pathways. Indeed, VDD is also associated with muscle fiber atrophy, an increased risk of chronic musculoskeletal pain, sarcopenia and associated falls [108] in patients with sarcopenic obesity and alcohol use-related disorders [73].

Tocopherols (vitamin E) are lipophilic molecules that can be found in seeds and nuts, olives and extra-virgin olive oil, avocadoes and whole cereal germs. Together with carotenoids, they display antioxidant properties and are the major protective agents against free radical-mediated liver damage, in particular lipid peroxidation. In patients with cholestatic liver diseases, particularly, low levels of circulating vitamin E were detected [109], and a selective hepatic depletion of carotenoids and tocopherols was detected in cirrhotic patients of mixed etiologies compared to controls, even in the presence of normal serum levels [110]. Contrasting results have emerged regarding the correlation between serum levels of vitamin E and muscle strength and physical function, probably partly due to the different demographic characteristics of the populations examined and the diverse methods to assess food consumption [111,112,113].

Vitamin K is a fat-soluble vitamin that naturally occurs in two forms, as vitamin K1 (phylloquinone) and vitamin K2 (menaquinone). K1 is the principal dietary form and can be found in green vegetables, kale, broccoli, cauliflower, cabbage or supplements, whereas K2 is produced by bacteria in the gut but can also be found in fermented soy and animal products. Vitamin K acts as cofactor in the carboxylation of many coagulation factors in the liver and, by stimulating vascular smooth muscle differentiation, it improves muscle perfusion, enhances skeletal muscle mitochondria functions and may play a possible favorable role in sarcopenia [114]. When supplies are low, vitamin K is preferentially used for the activation of coagulation factors in the liver. Indeed, observational studies demonstrate that prolonged reduced vitamin K status is associated with increased arterial stiffness and vascular calcification, a higher risk of fatal and non-fatal cardiovascular events, osteoporosis and sarcopenia [115].

*Group B vitamins:* Water-soluble group B vitamin deficiency is frequent in chronic liver disease due to reduced hepatic reserve, hepatocyte dysfunction and an inadequate and nutritionally poor diet, especially in cases of alcohol abuse [101]. Deficiencies of vitamins B1 (thiamine) and B3 (niacin) are associated with neuro-muscular alterations, such as muscle weakness and fatigue, whereas vitamin B6 (pyridoxine) deficiency is associated with effects on the peripheral nervous system and with loss of motor function [116]. Thiamine is contained in both animal (mainly liver, kidney and heart) and plant foods. A deficiency of this vitamin, especially under conditions of malnutrition and alcohol abuse, determines a vitamin B1-dependent enzyme dysfunction, with a consequent increase in reactive oxygen species and mitochondrial damage, ending in neuromuscular injury [116,117]. If severe, thiamine deficiency, through influencing cardiovascular, nervous and immune systems, can lead to life-threatening clinical syndromes such as beriberi and Wernicke–Korsakoff encephalopathy, which require emergency parenteral administration of high doses of thiamine [118]. Mild thiamine deficiency can instead induce mild cognitive impairment, loss of lean mass and strength, with onset of tremors and muscle weakness, predisposing to frequent falls. A clinician must be ready to identify and treat these symptoms promptly to prevent the development of dramatic conditions. Vitamin B9 (folate), which is mainly found in green leafy plants, seems to be indirectly related to the onset of sarcopenia because of increased blood levels of homocysteine in cases of deficiency, whether singularly or in combination with other micronutrients (such as B6 and B12 vitamins and choline). Some studies performed in older adults correlated high homocysteine levels with a loss of muscle mass and strength through an increased ROS-mediated mitochondrial damage, together with a reduction in muscle blood supply due to lower nitric oxide levels, resulting in loss of muscle mass, less muscle regeneration and loss of strength and endurance [116,119]. This hypothesis is corroborated by some observational studies that found an association between the presence of sarcopenia and lower intakes of folate, vitamin B6 and vitamin B12 [120,121]. Vitamin B12 is found in animal products (mainly eggs and dairy products) and, being mainly stored in the liver, its levels are reduced in liver cirrhosis. A deficiency of this vitamin can be associated with the onset of sarcopenia, either directly, as a result of degeneration and demyelination of the posterior and lateral tracts of the spinal cord, or indirectly, by leading to a worsening of cognitive status, with mood deflection and increased sedentary behavior [122].

#### 4.5.2. Minerals

*Zinc*: Zinc plays a pivotal role in most metabolic and immunologic pathways, being an essential cofactor for the catalytic domain of more than 300 enzymes. Zinc deficiency characterizes advanced stages of liver disease, being detected in almost 50% of cirrhotic patients and in up to 90% of those with albumin serum levels < 3.5 g/dL [123]. In cirrhotic patients, many mechanisms contribute to reduced zinc levels, namely, nutritional deficiency, decreased intestinal absorption, porto-systemic shunts, decreased hepatic extraction and, most of all, hypoalbuminemia, as albumin-free zinc is lost into the urine. In addition, muscle catabolism, together with the use of diuretics that inhibit renal tubular reabsorption of zinc, increase its renal excretion [123,124,125]. Zinc deficiency can cause a wide range of symptoms, including appetite loss, body hair loss, impaired taste and smell, atrophy of testis, cerebral and immune dysfunction and impairment of drug excretion ability. Zinc also plays a key role in the regulation of insulin secretion and activation, and its deficiency contributes to impaired glucose tolerance [125]. In the liver, zinc exerts certain functions that are not replaceable: the urea cycle, through which ammonia is converted into the non-toxic metabolite urea and is catalyzed by zinc-containing enzymes; if zinc deficiency occurs, ammonia processing is reduced, thus increasing the risk of toxicity [124]. Furthermore, zinc depletion contributes to the development of hepatic fibrosis by triggering collagen synthesis by stellate cells and altering the degradation of the extracellular matrix by zinc-dependent enzymes. Indeed, some studies demonstrated that zinc supplementation can improve hepatic fibrosis [126]. Finally, zinc deficiency has been associated with liver carcinogenesis and has been identified as an independent prognostic factor in patients with early HCC due to viral hepatitis treated curatively [127,128]. Zinc is also crucial in the crosstalk between the liver and muscles, and low zinc levels independently predict sarcopenia and frailty in patients with liver cirrhosis due to a mechanism mediated by the increase in circulating ammonia [125,129]. In obesity, zinc deficiency is associated with inflammation, oxidative stress and both lipid and glucose metabolism impairment [130], with zinc supplementation shown to improve body weight management [131].

*Magnesium*: Magnesium is one of the most prevalent intracellular cations and is involved in a wide range of biological processes and pathways that influence muscle function, such as transmembrane transport and energy metabolism, being essential for both muscle relaxation and contraction. Higher intakes of magnesium have been positively correlated with appendicular muscle mass, fat-free mass and muscle strength in young and older adults [132], and a number of observational studies showed sarcopenic older adults to have lower magnesium intake compared to non-sarcopenic subjects [133]. Reduced plasma levels of magnesium are frequently detected in obesity [134], and obesity-related hypomagnesaemia has been associated with insulin resistance, atherosclerosis, myocardial infarction and hypertension. On the other hand, a study performed on patients with morbid obesity undergoing bariatric surgery demonstrated an increase in magnesium and zinc levels associated with weight loss and, in the case of magnesium, to better glycemic control [135]. When it comes to liver disease, magnesium deficiency has been undoubtedly proven only in patients with alcohol-related etiology. In addition to malabsorption, a combination of elevated aldosterone, loop diuretics and indirect alcohol effects on renal tubules determine an excessive renal loss [136]. However, if the association between magnesium serum levels and cirrhosis severity is still controversial, a recent report on cirrhotic patients undergoing liver transplantation demonstrated a reduced intrahepatic magnesium content together with an overexpression of TRPM7 (a magnesium influx coenzyme involved in inflammation) in hepatocytes, as compared to deceased donors with a healthy liver. This finding suggests a possible involvement of intrahepatic magnesium imbalance in hepatocyte injury, as the MELD-Na score was correlated inversely with the intrahepatic magnesium content and directly with TRPM7 hepatocyte expression [137].

*Selenium*: Selenium displays direct antioxidant properties, and it is necessary for the adequate function of the immune system. Selenium deficiency has been related to the severity of hepatic fibrosis and found as one of the factors contributing to insulin-resistance in patients with chronic hepatitis C [138]; in addition, low levels of selenium have been associated with hepatocyte ballooning in alcohol-related liver disease [139].

#### 4.5.3. Other Nutrients

*Carnitine*: Carnitine is a quaternary ammonium compound required for the transport of long-chain fatty acids into mitochondria for energy production, but it is also involved in gluconeogenesis, the urea cycle, the glycolysis system and the tricarboxylic acid cycle. As a result, carnitine improves inflammation, oxidative stress and biomembrane function and contributes to skeletal muscle protein homeostasis by regulating both protein synthesis and breakdown [140]. Carnitine is found in animal products such as meat, fish, poultry and dairy products, but it is also synthesized by the liver and kidneys to be stored in the muscles. In sarcopenic cirrhotic patients, the risk of carnitine deficiency increases, as there is an association between impaired hepatic biosynthesis and reduced muscular storage [141]. As quantifying the prevalence of carnitine deficiency in cirrhotic patients may be difficult because serum levels do not reflect muscle stores, its deficiency can be diagnosed “ex adiuvantibus” in some cases, after significant improvement in clinical symptoms and signs as a result of carnitine supplementation [140].

### 4.6. Dysbiosis

The gut–liver axis consists of a very close and reciprocal interplay between the gut (and its microbiota) and the liver. The gut–liver route exploits the portal vein system to transport gut-derived environmental materials (as dietary, microbiological, toxic, etc.) directly to the liver, whereas the liver–gut way uses the biliary tract to secrete liver-derived products (as bile components and antibodies to shape the microbiota composition) into the gut. The condition of eubiosis (physiological distribution of microbial communities) is critical to maintain the homeostasis of the gut-liver axis and the disruption of this axis is involved in the pathogenesis of many non-communicable chronic diseases, especially those involving the liver [142].

In cirrhosis, severe modifications in gut microbiota, with an imbalance favoring pathogenic species (a condition known as dysbiosis) [143], are associated with portal hypertension and the alteration of the intestinal barrier due to congestion of the intestinal mucosa and the collapse of tight junctions, leading to an increased intestinal permeability and bacterial translocation. Filtering all the products of the digestive system, the liver (especially if already suffering from a chronic disease), is overwhelmed by the increased inflow of bacteria and their metabolites and reacts by activating the inflammatory cascade, which exacerbates liver damage. So, on one hand liver dysfunction worsens dysbiosis by a biliary ineffective control on microbiota composition, and on the other hand, dysbiosis also worsens liver function by providing the basis for a chronic low-grade inflammatory condition [142,143].

Dysbiosis in cirrhosis is characterized by a selective reduction in bacterial species that produce short chain fatty acids (SCFA), metabolic modulators that play a non-tissue-specific trophic role, especially for the intestinal barrier and the skeletal muscle. In addition, dysbiosis determines a less effective conversion of primary to secondary bile acids, whose receptors, once activated by their ligands in experimental settings, mediate muscle hypertrophy and cell differentiation; as a result, dysbiosis increases protein catabolism and contributes to skeletal muscle mass loss in animal models [144,145]. Indeed, the severity of sarcopenia has been associated with the grade of dysbiosis even within a human cirrhotic population [13]. On the other hand, dysbiosis with similar alterations in microbiota composition (diversity reduction, with an increase in Firmicutes/Bacterioidetes and a reduction in A. muciniphila) has also been demonstrated in obesity, where the systemic inflammation activated by dysbiosis and a leaking gut barrier lead to inflammation in metabolic tissues [146].

## 5. Sarcopenic Obesity Diagnosis in Cirrhosis

Standard diagnostic criteria for sarcopenic obesity are missing. Since its first definition in 2000, it has been described as the co-presence of sarcopenia and obesity [147], with an overall altered body composition, characterized by an increase in body fat together with a reduction in skeletal muscle mass. Nevertheless, in recent years a more functional definition of sarcopenia (and thus of sarcopenic obesity) has been proposed [9], as it has been demonstrated that muscle strength is more accurate than muscle mass in predicting adverse outcomes [148,149,150,151].Recently, the European Society for Clinical Nutrition and Metabolism (ESPEN) and the European Association for the Study of Obesity (EASO) proposed a multi-step diagnostic procedure for assessing sarcopenic obesity in the general population [40]. According to ESPEN and EASO, people are likely to have sarcopenic obesity if they simultaneously present an elevated body mass index (BMI) or waist circumference and clinical symptoms/risk factors for sarcopenia or a positive score at validated self-reported questionnaires, such as the SARC-F. This simple screening tool evaluates five components: strength, assistance with walking, rising from a chair, climbing stairs and falls. The score ranges from 0 to 10, with 0 to 2 points for each component; a total score equal to or greater than 4 is predictive of sarcopenia and indicates poor outcomes [152]. A positive screening result needs to be followed by the diagnostic phase, for which ESPEN and EASO recommend assessing skeletal muscle functional parameters and body composition. The first can be evaluated as hand-grip strength, knee extensor strength or chair-stand test. Although various studies suggest the necessity to adjust skeletal muscle strength to body weight/BMI [153,154], there is currently no sufficient evidence and there are no clear cut-offs [155]. Body composition is assessed as the distribution of fat mass and skeletal muscle mass, adjusted for body size in different ways, namely, height squared, body weight or body mass index [156,157,158]. Muscle quantity can be reported as total body skeletal muscle mass (SMM), appendicular skeletal muscle mass (ASM), or the cross-sectional area of specific muscle groups or body locations. ESPEN, EASO and AWGS2019 suggest the use of dual-energy X-ray absorptiometry (DXA) or bio-electrical impedance analysis (BIA) for body composition evaluation. Notably, body mass normalization can be unreliable in cases of a significant increase in body water (e.g., ascitic effusion in cirrhotic patients), and both DXA and BIA measurements can be influenced by the hydration status of the patient. According to ESPEN and EASO, the diagnosis of sarcopenic obesity is confirmed in the presence of both altered body composition and impaired skeletal muscle functional parameters [40].

For the general population, as well as in patients with cirrhosis, most of the latest literature suggests that sarcopenia should be defined as both muscle mass and strength loss or reduced performance [101,159]. As for obesity, its traditional definition based on body mass index (BMI) may be particularly incorrect due to fluid retention that is typical in the decompensated stages of liver disease [160]. In the absence of imaging tools, a BMI corrected for ascites [160] is the easiest for use in clinical practice and most consistent with the non-cirrhosis literature [161]. In cases of fluid retention, the BMI needs to be calculated using the patient’s dry weight, commonly estimated using either the post-paracentesis body weight or the weight recorded before fluid retention, if available, or by subtracting 5%, 10%, and 15% of the actual weight in the presence of mild, moderate, or severe ascites, respectively. An additional 5% is subtracted for peripheral edema, if present [14,162].

According to the diagnostic procedure proposed by ESPEN and EASO, all patients with chronic liver disease (especially NASH and cirrhosis) and elevated BMI or waist circumference are at risk of sarcopenic obesity and should be therefore tested for muscle strength and body composition [14]. As in the general population, CT and MRI represent the gold standard to assess body composition [163,164,165], and in patients with decompensated cirrhosis, they are particularly useful since they allow muscle assessment, including cross-sectional area measurement and muscle attenuation [50], without being biased by fluid overload.

In the 2021 practice guidance, the American Association for the Study of Liver Diseases (AASLD) endorsed the skeletal mass index (SMI), assessed via CT image analysis and calculated as the total skeletal muscle area at L3 vertebra normalized to height squared, as the most consistent and reproducible method to quantify muscle mass in patients with cirrhosis [166]. Because of the risk of exposure to radiation, AASLD and EASL don’t recommend the use of abdominal CT solely for the purpose of muscle mass measurement but suggest muscle mass quantification whenever an abdominal CT is obtained as part of clinical care or in patients in whom the assessment of muscle contractile function is not practical or feasible (e.g., acutely ill patients). According to AASLD, sarcopenic obesity should be defined as the coexistence of low sex-adjusted SMI and BMI ≥ 25 kg/m^2^, suggesting a stricter BMI cut-off for obesity when compared to the definition provided by the ESPEN and EASO for the general population [40], as well as the definition given by EASL for chronic liver disease (obesity if BMI ≥ 30 kg/m^2^) [1]. In the literature, only few studies used DXA to diagnose sarcopenia in patients with cirrhosis due to concerns of overhydration influence on muscle mass estimation. Recently, more attention has been given to arm lean mass assessment rather than leg lean mass and total appendicular lean mass since the former appears to be more accurately associated with cirrhosis severity [167], as upper limbs are less involved in gravitational fluid retention. Recent Asian studies have shown promising results in body composition evaluation with BIA in MASLD patients with sarcopenic obesity [168] and good concordance between BIA and DXA in estimating fat mass and free-fat mass in patients with cirrhosis and a maximum mild grade of ascites [169]. These findings are consistent with the EASL suggestion to consider DXA or BIA for sarcopenia evaluation in the absence of fluid retention [1].

### Other Evaluations in Patients with Cirrhosis: Malnutrition and Frailty

Malnutrition is defined as a clinical syndrome that results from “an imbalance (deficiency or excess) of nutrients that causes measurable adverse effects on tissue/body form (body shape, size, composition) or function, and/or clinical outcome” [170]. Malnutrition represents a spectrum of nutritional disorders across the entire range of body mass index (BMI), from underweight to obese, leading to adverse physical effects, which, in patients with cirrhosis, are commonly manifested as frailty or sarcopenia [166]. The ESPEN and EASL guidelines recommend the Royal Free Hospital-Nutritional Prioritizing tool (RFH-NPT) to identify malnutrition risks in patients with liver disease [1,171]. RFH-NPT classifies patients into low-, medium- or high-risk categories and, according to EASL, patients who are at high-risk of malnutrition should undergo a detailed nutritional assessment for the diagnosis of malnutrition every 1–6 months [1], including an evaluation of muscle mass (presence/absence of sarcopenia), the use of global assessment tools to determine nutritional status and a detailed dietary intake assessment [1].

Frailty can be defined as the loss of functional, cognitive and physiologic reserve, leading to a vulnerable state, and may be considered a form of nutrition-related disorder [1]. Tools to assess frailty as a multidimensional construct (e.g., global frailty) or its individual components (e.g., physical frailty, disability, functional status) have been developed in patients with cirrhosis. Among them, the liver frailty index (LFI) measures physical frailty using the combination of three objective, performance-based tests of physical function: grip strength, chair stands (CST) and balance tests and defines patients as robust, prefrail and frail (liver frailty index ≥ 4.4) [172]. Of note, a frail condition has been recently associated with mortality in patients with cirrhosis independently from the presence of major liver failure complications [173].

## 6. Nutritional Interventions in Cirrhotic Patients with Sarcopenic Obesity

**Figure 2:**
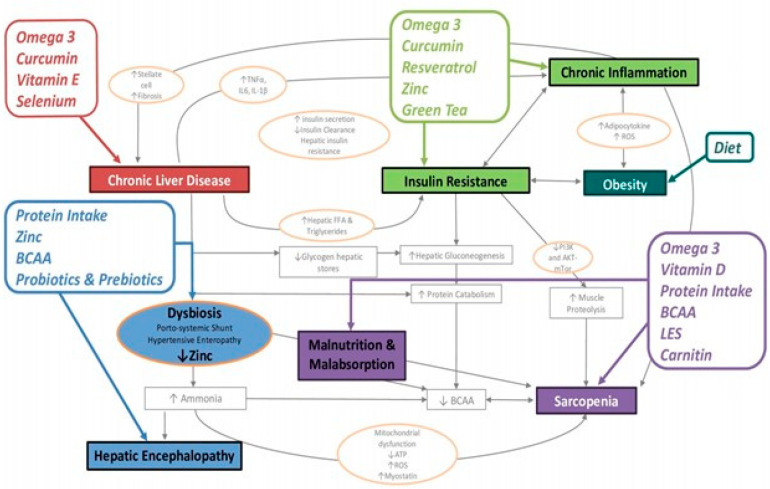
BCAA: Branched-chain amino acids; LES: late evening snack; diet: moderately hypocaloric and normo-hyperproteic.

In order to correctly set up a personalized nutritional intervention, it is necessary to define the single patient’s needs in terms of energy, macro- and micronutrients. Listed in Figure 2 are the main nutritional interventions that we can implement in cirrhotic patients with sarcopenic obesity, based on the pathophysiological mechanisms on which they act.

### 6.1. Energy and Macronutrients

In general, the caloric requirement of a patient with compensated cirrhosis is comparable to that of the general population. This similarity exists because cirrhotic patients often display a reduced level of physical activity, resulting in lower energy costs. Nevertheless, in about one-third of patients with cirrhosis, a hypercatabolic condition develops, characterized by a reduction in protein synthesis and an increase in proteolysis to provide free amino acids to support gluconeogenesis, which is a high energy expenditure process [174]. The state of accelerated starvation is further exacerbated during the acute and chronic decompensation phases of the disease, particularly in cases of severe portal hypertension and hepatic encephalopathy or refractory ascites subjected to maximal diuretic therapy and paracentesis. Frequent reassessment of nutritional status and resting energy expenditure (REE) is therefore highly recommended in these patients, especially in those meeting criteria for malnutrition or sarcopenia at baseline, and whenever significant changes in clinical condition develop [166]. REE can be evaluated by indirect calorimetry (gold standard) or, if this technique is not available, it can be roughly estimated by predictive equations, such as Harris–Benedict. Based on consolidated data reporting in cirrhotic patients, a REE of 28–38 kcal/kg/day [175,176], and considering that energy supply needs to balance total energy expenditure (TEE), including REE, food-related thermogenesis and energy expenditure related to physical activity, current EASL, ESPEN and AASLD nutrition guidelines agree on recommending, for normal-weighted patients with compensated cirrhosis, an intake of at least 35 kcal/kg of body weight with a protein intake of 1.2–1.5 g/kg/day to guarantee metabolic homeostasis. In patients with fluid retention, body weight needs to be corrected by evaluating the dry weight as previously reported [1,166,171].

In obese subjects without hepatic disease, as well as in obese cirrhotic patients, weight loss has been proven to be beneficial, with a weight decrease of 5–10% associated with a reduced rate of liver disease progression [32,177]. Even though to date no cirrhosis-specific intervention trials have been conducted in patients with sarcopenic obesity, the dietary approach to achieve weight loss without compromising protein stores in cirrhotic patients with obesity, as suggested by the abovementioned international guidelines, should be based on a tailored and moderately hypocaloric diet (500–800 kcal/day of deficit or alternatively a daily intake of 20–25 kcal/kg of actual dry weight for those patients with BMI > 35–40, with an adequate protein intake of 1.5 g per kg of ideal body weight (defined as the dry body weight at a BMI of 25 kg/m^2^) [1,166,171], which could be further increased in cases of sarcopenic obesity and/or malnutrition. Indeed, some studies have reported positive outcomes with a protein intake even >2 g per kg of ideal body weight, especially in decompensated disease (such as bleeding or infections) or after surgery, when protein needs are particularly high [178]. In patients with ascites, more concentrated high-energy formulae should be preferred to avoid liquid overload.

The best protein source (animal, including dairy, or vegetable) for cirrhotic patients is still debated so, to date, it is recommended to maintain adequate protein intake from diverse range of sources, including vegetable and dairy products, when possible [166]. Despite a lack of a strong evidence in favor of a strictly vegetarian diet in cirrhotic patients, the general belief is that a prevalence of vegetable proteins may be beneficial, since they are rich in branched-chain amino acids (BCAA: valine, leucine and isoleucine) compared to animal proteins, and BCAA remove one mole of ammonia per mole of BCAA. The proteins of vegetable and dairy origin may improve the nitrogen balance and, if well tolerated, they can be provided without constraints. Vegetable and dairy (especially whey) protein-rich diets were linked to higher skeletal muscle mass and reduced sarcopenia in various series of elderly patients affected by cirrhosis or sarcopenic obesity. Intestinal microbial diversity is influenced by dietary protein source and its amino acid composition. A more favorable gut microbiome is associated with regular ingestion of vegetal and fermented cheese and whey proteins [179,180,181].

Thanks to their high content of fiber, vegetable foods may also influence intestinal transit and the ISHEN recommends a daily ingestion of 25–45 g of fiber in cirrhotic patients due to its ability to eliminate the nitrogen products of the colon and to reduce the degree of the patient constipation [178].

In the case of insufficient alimentary protein intake, BCAA or leucine-enriched BCAA supplementation can be considered. The latter shows a stimulatory effect on mTORC1 in the skeletal muscle, even in patients with alcohol-related cirrhosis, and on the production of hepatocyte growth factor, a pleiotropic ligand with mitogenic activity secreted by hepatic stellate cells that is involved in the regenerative process of the liver [182,183]. Since a lack of BCAA can accelerate molecular protein catabolism, decreased albumin synthesis and hyperammonemia with hepatic encephalopathy, many studies aimed at investigating if enhancing BCAA availability may be beneficial for muscle and brain metabolism in liver cirrhosis, a condition where an imbalance between aromatic amino acids (AAA: phenylalanine, tyrosine and tryptophan) and BCAA in favor of the first fraction is common. Indeed, a previous Cochrane meta-analysis demonstrated the beneficial effects of BCAA supplementation on hepatic encephalopathy, both minimal and overt, probably thanks to multiple mechanisms (increase of muscle ammonia detoxification, improvement in brain energy metabolism, reduction in the AAA cerebral flow) [184] but the effects of BCAA supplementation on other aspects of advanced liver disease are less clear, mainly due to the high heterogeneity of the protocols (period and time of supplementation) and of the populations analyzed. In general, long-term supplementation of BCAA to cirrhotic patients significantly increases event-free survival and overall survival, despite equivocal results regarding liver function and sarcopenia parameters. However, no serious adverse events were reported for BCAA supplementation, even in advanced liver disease and, therefore, though not recommended beyond the adequate protein intake from different sources, BCAA supplementation can be useful to reach the protein intake goal in the case of insufficient dietary ingestion [185,186,187,188]. β-hydroxy-β-methylbutyrate (HMB) is an active metabolic derivative of leucine, which is synthetized in the liver, and appears to be even more effective than leucine in promoting protein synthesis, tissue repair and aerobic performance, while inhibiting proteolysis and autophagy in muscle cells. A few recent studies evaluated the effects of HMB supplementation before and after liver transplantation, with highly different protocol designs and conflicting results [189]. L-carnitine supplementation alone, or in addition to BCAA, has been reported in some small studies to determine dose-related lowering of ammonia levels, and thus to have a beneficial effect on skeletal muscle mass in cirrhotic patients, but the evidence provided so far is largely insufficient to suggest its regular supplementation in clinical practice [190].

Enteral nutrition with oral supplementation, initially only during the night period and, if necessary, also during the daytime, should be initiated as soon as possible (first 24–48 h) in patients unable to ingest a minimum of 1 g/kg (weight) of daily proteins. Tube feeding and parenteral nutrition may improve nutritional status, liver function, reduce complications and increase survival, These options may be considered as secondary choices for nutritional support in severely ill patients with impaired chewing and swallowing who are not able to eat enough and safely [171].

Of note, the combined approach of a hypocaloric/high-protein diet seems to be more effective in the prevention than in the treatment of sarcopenic obesity, especially in obese and physically limited older adults, where the results on muscle mass and performance are inconsistent, making the diagnosis and setting up of personalized dietary treatment even more urgent in frail patients at high risk of sarcopenia [191,192,193].

As for the remaining macronutrient composition of the diet, a Mediterranean approach is recommended due to its beneficial effects on body weight, insulin sensitivity and hepatic steatosis and fibrosis, even without weight loss [171].

Carbohydrates must represent the basis of the diet in cirrhotic patients and should cover 50–60% of non-proteic daily needs [194], with a preference for foods rich in complex carbohydrates [195]. Sustaining pro-inflammatory processes by consuming ultra-processed food, typically energy-dense and low in nutrient content, should be avoided and, indeed, a reduced daily intake of simple sugars (sugary sweets and added sugars, jam and honey) has been recently associated with a MELD improvement in cirrhotic patients, especially in those with visceral adiposity [196].

Compared to carbohydrate and protein, lipid metabolism seems to be less altered in patients with liver cirrhosis so, in the absence of specific suggestions, it is recommended to consider the lipid calories distribution as reported in the Mediterranean diet, with most of the energy deriving from unsaturated and polyunsaturated fats and less than 10% of the total energy intake coming from saturated fats [197]. In patients with steatorrhea, the diet content of long chain fatty acids should be reduced at the expense of medium and short chain fatty acids. Some patients, such as those with alcohol-related diseases, may require supplemental pancreatic enzymes due to the presence of pancreatic insufficiency [198].

Omega-3 fatty acids are polyunsaturated fats (PUFAs), including docosahexaenoic acid, eicosapentaenoic acid and docosapentaenoic acid, and are mainly contained in fish meat and oils, eggs, seafood and vegetable oils (extra-virgin and sesame seeds). PUFAs display immune-regulatory and probiotic properties, and recently, their supplementation has been associated with protein metabolism and insulin-resistance improvement. Long-term fish oil administration can enhance the anabolic stimuli from substrates, hormones and physical activity in skeletal muscle cells, and some studies have demonstrated that linolenic acid improves sarcopenia in an animal model by restoring mitochondrial function [199]. In clinical series of ageing adults, omega-3 intake has been positively associated with higher appendicular skeletal muscle mass index and a lower incidence of sarcopenia due to pleiotropic effects, including anti-inflammatory properties, muscle anabolic effects, through the activation of the mTOR signaling, and a reduction in insulin resistance [200,201]. In particular, supplementation with fish oil appears to enhance the neuromuscular response to the anabolic stimulus from training, potentiating muscle strength and physical performance in sarcopenic older women [202]. Especially in patients with alcohol-related steatotic liver disease, omega-3 fatty acids from fish oil are useful in reducing lipid accumulation in the liver and membrane lipid peroxidation. In animal models, administration of unsaturated fatty acids preserves mitochondrial function by reducing oxidative stress [203]. In human trials, omega-3 fatty acid supplementation failed to modify the histological features of MASLD/MASH, despite a significant reduction in serum liver enzymes and triglycerides, liver fat content and steatosis scores, so ESPEN suggests to limit their use in overweight/obese patients with chronic liver disease to improve the serum lipid profile [204,205].

### 6.2. Late Evening Snack

As cirrhosis is characterized by a state of accelerated starvation, the nutritional goal is to distribute the nutrient and caloric intake in small and regular meals throughout the day, every 3–6 h (the so-called “spread diet”), with a late evening snack (LES), to prolong the postprandial period characterized by a suppression of protein degradation in favor of synthesis stimulation [1,166,171]. The “spread diet” was associated with a higher protein synthesis rate, leading to greater muscle strength, better physical performance and increased skeletal muscle mass even in older adults without liver disease; in addition, some preliminary data suggest that increasing the number of meals per day may stimulate the overall satiety, thus reducing the obesity risk [192].

The last meal of the day should be consumed before bedtime (LES) in order to minimize night starvation to no longer than 6 h. In cirrhosis, due to the reduced glycogen storage, the liver starts to convert amino acids coming from the skeletal muscle into glucose to rebalance glycemia after a few hours of starvation. This typically occurs between the end of supper at dinner and the beginning of breakfast in the morning, a condition which is observed in healthy individuals after a fasting period of roughly 3 days [206]. LES, compared to daytime supplementation, was demonstrated to be the best option to improve the nutritional status in cirrhotic patients, with beneficial effects on hepatic biochemical parameters, including albumin, ammonia and prothrombin time, as well as on clinical events, such as the development of ascites and encephalopathy, resulting in an improved overall survival in some series [207,208,209,210].

Though all clinical guidelines agree on recommending a late evening snack, there is no consensus on the optimal meal composition. Several studies have investigated different LES formulations, including liquid nutritional supplements, snacks based on complex carbohydrates (e.g., bread and jam, a rice ball), complex carbohydrate and protein and BCAA-enriched supplements, with a mean of 200–250 kcal and approximately 13.5 g of proteins [211]. In cirrhotic patients, a LES containing a combination of complex carbohydrates and proteins reduces lipid oxidation, improves nitrogen balance, reduces skeletal muscle proteolysis, increasing muscle mass, reduces hepatic encephalopathy and improves the quality of life, though it has no clear effect in reducing mortality or the need for liver transplantation [207,208]. What is more, a recent meta-analysis demonstrated that LES, irrespective of its composition, besides improving malnutrition, also helped to maintain glucose homeostasis in diabetic cirrhotic patients [212]. Foods with high caloric content (at least 50 g of carbohydrates) and enriched with BCAA (leucine, isoleucine and valine) should theoretically be preferred because, if eaten at night, BCAA are used first for protein synthesis, while if administered during daytime, they are preferably used as an energy source [213,214]. In clinical practice, LES based on BCAA supplementations are rarely used due to their poor palatability and high cost. In general, providing variety with night meals appears to be effective, provided that they contain a reasonable combination of complex carbohydrates and proteins. Importantly, the meal composition needs to be tailored according to patients’ preferences and comorbidities (for example reflux complaints) in order to reach the highest compliance possible [195], as it was recently proposed in a practical chart menu [215].

### 6.3. Micronutrients

Malabsorption causes high rates of multiple micronutrient deficiencies in patients with liver cirrhosis due to a combination of pathological mechanisms (bacterial overgrowth, portosystemic shunting, protein-losing enteropathy, gastrointestinal dysmotility and intestinal edema). Fat-soluble vitamins malabsorption is exacerbated by the reduced excretion of bile salt in cholestatic diseases [101,216] and, in cases of concomitant obesity, their levels are further reduced due to the sequestration and altered metabolism in visceral adipose tissue.

Due to the high prevalence of general malabsorption, though in patients with preserved oral intake, there is little consensus regarding the widespread use of multivitamins or other micronutrient supplements, ESPEN and EASL guidelines agree that, besides treating clinically suspected or confirmed deficiencies following accepted general recommendations and common practice, empirical daily supplementation of water-soluble vitamins and minerals should be considered for all patients with advanced disease, as it is highly cost-effective when comparing the low cost of supplementation to the risk of nutritional deficiencies and the costs of specific nutritional evaluations [1,217].

Table 1 summarizes normal plasma levels, recommended daily allowance and schedule supplementation of different micronutrients in patients with chronic liver disease.

Hereafter, we will provide detailed information only on those individuals with available data on specific supplementation in cirrhosis and/or sarcopenic obesity.

#### 6.3.1. Vitamin D

Despite the broad evidence of the high frequency of VDD and its association with the severity of liver disease and poor prognosis [102,103,104], the efficacy of vitamin D supplementation in patients with liver disease has not been demonstrated convincingly, probably due to highly different study designs (selected populations, baseline vitamin D levels, laboratory and clinical endpoints). Therefore, a Cochrane review in 2017 concluded that vitamin D supplementation had neither beneficial nor harmful effects on all-cause mortality in adults with liver disease, lacking convincing evidence for a therapeutic impact in this setting [218]. Updated ESPEN and EASL guidelines conclude that, in patients with liver disease, vitamin D supplementation has no proven benefit aside from correcting a deficiency state, similar to the general population. Plasma vitamin D levels should be assessed in all patients with liver disease, especially in those with steatotic, advanced or cholestatic disease, and supplementation should be prescribed whenever the levels are under the deficiency threshold, until reaching serum levels above 75 nmol/L or 30 ng/mL. No specific dosage is recommended, though the most frequent schedules of administration are 800–2000 UI/day [1,171]. In sarcopenic older adults, vitamin D supplementation at daily doses of 800–1000 UI improved several sarcopenic parameters [219], and the benefits of simultaneous Ca and vitamin D supplementation in preventing bone loss, reducing bone turnover and non-vertebral fractures, have been clearly confirmed in postmenopausal women [220]. According to a short-term longitudinal study recently conducted in monozygotic twins, calcitriol administration (2000 UI/day for two months) determined a significant decrease in total body fat, together with an increase in gynoid lean mass [221]. A great body of evidence on interventional studies with vitamin D supplementation is available but no definite conclusions can be drawn on its effect on muscle mass and function. Significant results were obtained in populations with lower baseline vitamin D levels [118,222] or with the combination of vitamin D supplementation and resistance training [223]. As patients eating a high protein diet can lose an excessive quantity of calcium in their urine, and even more if they are treated with diuretics [224], it may be suggested to these patients to enhance the consumption of selected foods with low levels of oxalic acid (e.g., bananas, blueberries, apples, broccoli, cabbage, white rice, eggs, meat, fish, yogurt, cheese, milk, fruit juice) and phytic acid (food processed by several pretreatment methods, such as fermentation, soaking, germination and enzymatic treatment), which can contribute to enhanced calcium incorporation into the skeleton.

To date, there are not specific guidelines or recommendations regarding vitamin D supplementation in cirrhotic patients with sarcopenic obesity, but it is reasonable to encourage them to increase their daily exposure to sunlight, ideally 30 min during the daytime, as obesity determines a lower increase in circulating concentrations of vitamin D after irradiation [107,225]. If patients like and tolerate them, they can be encouraged to include in their diet foods naturally rich in vitamin D, such as egg yolks, oily fish and dairy products from animals raised outdoors.

#### 6.3.2. Vitamin E (Tocopherol)

As vitamin E displays fundamental antioxidant properties, and its depletion has been documented in cirrhotic patients, pre-clinical and clinical studies have been performed to evaluate the effects of its supplementation in this group of patients. A high dose of oral vitamin E supplementation (800 UI/day) was safely administered to obese non-diabetic MASH patients for 24 months, resulting in improvement in inflammation but not of fibrosis [226]. Indeed, according to ESPEN, vitamin E supplementation (800 UI/day) should be prescribed to all non-diabetic, non-cirrhotic, biopsy-proven NASH patients [171]. As cirrhotic subjects were excluded from this study, causing a lack further evidence in this specific subset of patients, current guidelines do not recommend vitamin E supplementation to cirrhotic patients with obesity, even when dysmetabolic liver disease is present [177]. This caution is justified by the fact that vitamin E toxicity can cause major bleeding events, whereas the possible association between vitamin E supplementation and an increased risk of prostate cancer has not been confirmed by a recent metanalysis [227]. Few data are available to evaluate the effects of antioxidant supplementation in sarcopenia, with overall inconsistent evidence [228]. As a simple supplementation approach, providing antioxidants (vitamin E in particular) appears not to be effective in improving muscle health. Nevertheless, cirrhotic patients with sarcopenic obesity should be encouraged to eat foods naturally rich in vitamin E, such as seeds and nuts, olives and extra-virgin olive oil, avocadoes and whole cereal germs, to reach the dietary reference intake for adults of 15 mg (22 UI)/day of alpha-tocopherol.

#### 6.3.3. Vitamin B1 (Thiamine)

Vitamin B1 requirement is set at 1.2 mg/day for men and 0.9 mg/day for women and can be generally satisfied by a various diet. Vitamin B1 deficiency, on the other hand, is common in patients with alcohol abuse and, given the potential life-threatening clinical syndromes caused by its severe deficit, thiamine and other vitamins of group B supplementation should be considered in all patients, especially in those who have alcohol-related disease or appear to be severely malnourished; being water-soluble, their long-term administration has been reported to be safe even at high doses [229]. Observational data suggest that vitamin B supplementation should be effective at promoting muscle health, but strong evidence coming from interventional studies is still lacking. In addition, due to challenges in interpreting and defining the roles of individual B vitamins in dietary datasets in which they are highly correlated, and indeed in a study on a cohort of older Dutch individuals, higher intakes of vitamin B6, B12 and folate were positively correlated with better functional scores [230].

#### 6.3.4. Zinc

In cirrhotic patients, zinc deficiency (<60 μg/dL) has been associated with insulin-resistance, hepatic steatosis, iron overload, skin and hair alteration, impaired night vision, altered wound healing, dysgeusia and hepatic encephalopathy due to the reduced activity of urea cycle enzymes [231,232]. Most studies on zinc supplementation in cirrhosis focused on its effects on hepatic encephalopathy. Despite the fact that some authors demonstrated an improvement in amino acids metabolism and hepatic encephalopathy [233,234,235,236,237], while a few studies reported a better prognosis due to lower frequency of liver decompensation and HCC development in cirrhotic patients with low zinc serum levels treated with supplementation [238,239], there is still no consensus on the dose and timing of supplementation. A particular case is represented by chronic alcohol intoxication, where low levels of brain zinc have been associated with an increased sensitivity to alcohol withdrawal-induced seizures, and zinc supplementation may alleviate general alcohol withdrawal symptoms [240]. Some studies in the literature have shown an improvement in dysgeusia and muscle cramps in cirrhotic patients who were supplemented with vitamin A and zinc [54]. Despite there being no published literature exploring the efficacy of zinc in cirrhosis with sarcopenic obesity, it can be postulated that, through ammonia-lowering effects, zinc supplementation may help to counteract sarcopenia in this specific context.

In general, as with other micronutrients, zinc supplementation should be provided at least until reaching normal blood levels, at a dose of 50 mg of elemental zinc (229 mg zinc sulphate) once daily [241].

#### 6.3.5. Sodium

Sodium restriction to 2 g/day in addition to a total salt intake of 5 g/day is recommended by ESPEN, EASL and AASLD to counteract fluid overload in cirrhotic patients, especially in cases of ascites and edema scarcely responsive to diuretic therapy [1,171,242]. However, the evidence regarding the beneficial effects of sodium restriction in cirrhotic patients is limited and conflicting, with one study reporting low plasma sodium levels, higher creatinine levels and higher time for ascites resolution in decompensated cirrhotic patients on a strict sodium-restricted diet [243]. Following sodium restriction is difficult in real life, as adherence to the prescription is poor, and a true low-salt diet may determine an overall lower dietary intake due to low palatability, so nutrition guidelines in frail sarcopenic cirrhotic patients suggest prioritizing the quantity and quality of the food at the expense of its sodium content [211]. As hyponatremia is common in patients with cirrhosis, careful monitoring of both sodium and water intake is required. Fluid restriction should only be recommended in severe hyponatremia (Na^+^ <120 mEq/mL) and is not indicated in compensated liver disease; hence, it is important to have a dietitian who can educate the cirrhotic patient to use different strategies to flavor or make low-salt foods more palatable [244].

#### 6.3.6. Carnitine

High-dose L-carnitine supplementation (2 g/day) displayed a positive impact on oxidative stress and inflammation, with an improvement in physical and mental fatigue, quality of life, nutritional status and sarcopenia in a cohort of centenarians [245] but also in patients with chronic diseases, including cancer and chronic hepatitis C infection [246]. In patients with liver cirrhosis, the supplementation of L-carnitine (1.5–2 mg/day), with or without co-administration of BCAA, has been associated with an improvement in muscle mass and function, cognitive deficit and electro-encephalogram alterations through a reduction in serum ammonia concentration [247,248,249,250]. In addition, L-carnitine therapy in a group of cirrhotic patients with advanced disease was reported to determine a change in energy expenditure, with a reduction in the oxidation of fats and proteins and an increase in that of carbohydrates and an overall decrease in inflammation markers [251].

**Table 1:** Normal plasma levels, recommended daily allowance (RDA) and supplementation schedule of different micronutrients in patients with chronic liver disease.

Micronutrient	Normal Plasma Levels	RDA	Schedule of Supplementation
Vitamin D	>50 nmol/L >20 ng/mL	400 IU/day	800–2000 IU/day in all patients with VDD [1,171]
Vitamin E		15 mg (22 IU) of alpha-tocopherol	800 IU/day in non-diabetic non-cirrhotic biopsy-proven NASH patients [171]
Vitamin B1		0.9 mg/day for women 1.2 mg/day for men	Consider water-soluble vitamin and mineral supplementation in all patients with advanced liver disease and alcohol abuse. Avoid manganese-containing formulations due to possible accumulation in basal ganglia structures of the brain [1,171]
Vitamin B6		1.1 mg/day for women 1.5 mg/day for men	
Vitamin B9		200 µg/day	
Vitamin B12		2–2.5 µg/day	
Magnesium	1.8–2.4 mg/dL	320 mg/day for women 420 mg/day for men	
Zinc	>60 µg/dL	11 mg/day for women 8 mg/day for men	zinc acetate 150–600 mg/d [234,235,238] zinc sulphate 200–600 mg/d [232,235] elemental zinc 20–50 mg/d [233,235,236]
Carnitine			1.5–2 mg/day in sarcopenic cirrhotic patients [247,248,249]

RDA: recommended daily allowance; IU: international unit. VDD: vitamin D deficiency. ALD: alcohol-related liver disease.

### 6.4. Nutraceuticals

#### 6.4.1. Polyphenols

Polyphenols are secondary metabolites of plants, with one or more phenolic rings, naturally found in vegetables and fruits. They display a wide spectrum of biological activities (antioxidant, anti-inflammatory, immune-modulatory, proapoptotic and antibacterial), and their use has been demonstrated to delay the aging process and to reduce the incidence of non-communicable chronic diseases, such as cardiovascular disorders, cancer, type 2 diabetes mellitus, neurological diseases, and osteoporosis [252]. Many polyphenols have been evaluated as possible nutraceutical interventions in liver diseases and sarcopenia [199,253]. We report here the main data regarding three of the most studied molecules in the metabolic context.

*Curcumin* is the main natural polyphenol found in the rhizome of *Curcuma* spp. Curcumin displays its wide immune-modulating and anti-inflammatory effects by inhibiting several cell-signaling pathways, including NF-kB, and by modulating intestinal microbiota in favor of high-butyrate producing bacterial species in human healthy subjects. Histological findings from rodent studies showed that curcumin and related substances ameliorated liver fibrosis by inhibiting hepatic stellate cell proliferation, collagen synthesis, and matrix metalloproteinase and reduced the number of necrotic cells in a dose-dependent fashion [254]. In a recent meta-analysis of Asian clinical trials conducted for 8–12 weeks involving supplementation with different forms of curcumin, small but significant reductions in AST and ALT levels (but not in cholestasis markers) were observed, together with a significant reduction in ultrasound-evaluated liver steatosis, BMI, waist circumference, fasting blood glucose and total cholesterol; no significant variation in liver fibrosis at transient elastography was demonstrated, but this parameter was evaluated only in two studies [255]. Recent murine data suggest a possible muscle-specific response to curcumin treatment, with prevention of muscle mass loss, reduction of age-related muscle force loss and overall reduction in mortality in old mice [256,257]. In two studies involving healthy elderly subjects, supplementation with two different formulations of an oral bioavailable form of curcuminoids for 3 months resulted in a significant increase in handgrip strength, weight-lifting capacity, and walking distance covered before feeling tired, without any adverse effects [258,259].

*Resveratrol* is a compound with various anti-inflammatory, vaso-protective and regulatory properties, detected in numerous plant species (within roots, stems, flowers, leaves, seeds and fruits), including blueberries, grapes (especially the skin), peanuts and cranberries. Preclinical studies have hypothesized that resveratrol plays a pleiotropic role in reducing liver fibrosis by inhibiting stellate cells, reducing oxidative stress, lipid peroxidation and iron overload, improving hepatic glucose metabolism and insulin activity and inducing hepatic cancer cells apoptosis in different in vitro and in vivo models. In addition, other interesting data suggest that resveratrol may prevent brain edema and neuroinflammation by protecting the blood–brain barrier and facilitating the maintenance of its integrity, with a therapeutic potential in hepatic encephalopathy, mainly through gut microbiome modulation [260]. Limited data from human-based studies reveal that resveratrol improves blood pressure, waist circumference, insulin sensitivity and fasting glucose levels in patients with type 2 diabetes mellitus and may improve inflammatory status in individuals with obesity [261]. A few randomized controlled trials focused on the hepatic effects of supplementation with resveratrol for 4–12 weeks in MASLD patients, demonstrating a reduction in aspartate aminotransferase, glucose, low-density lipoprotein cholesterol and hepatic fat content as compared to the placebo group [262]. Pre-clinical experiments also demonstrated positive effects on muscle mass and function (through an enhancement in muscle protein synthesis, a decrease in muscle protein degradation and an attenuation of skeletal muscle fibers atrophy) and an improvement in mitochondrial function and density. In a recent paper reviewing clinical studies, globally positive or partially positive effects were obtained on skeletal muscle health in adult patients displaying features of metabolic syndrome, despite great differences in study designs (such as the association with physical exercise), resveratrol administration schedules and reported outcomes [263].

*Green tea* is obtained from the leaves of Camellia sinensis and is a traditional drink used for its beneficial effects in cardiovascular and other chronic diseases. Green tea extract, enriched in bioactive molecules catechins (especially the most active compound epigallocatechin-3-gallate, EGCG), has been reported to counter insulin resistance and hypertension via its antioxidant and anti-inflammatory properties. Some experimental studies have demonstrated hepatoprotective properties of green tea in preclinical settings, and therapeutic and favorable effects of Camellia sinensis, such as reducing mortality, attenuating steatosis and reducing the incidence of primary liver cancer, have also been reported in human cohorts [264]. Due to its anti-inflammatory, anti-obesity, anti-diabetic and weight-reducing effects, also mediated by the modulation of gut microbiota [265], EGCG has been successfully tested in clinical trials on patients with features of metabolic syndrome [266]. Two clinical trials conducted on two cohorts of sarcopenic elderly men and women also demonstrated a positive additive effect of green tea extract on the outcomes of exercise in increasing leg muscle mass and function [267,268]. However, while green tea infusion is widely consumed and generally safe, green tea extracts have shown to have a hepatotoxic potential. Since the first report in 1999, many cases of liver injury related to the intake of different green tea extracts have been recorded, some of them with a positive rechallenge. The physiopathology of green tea inducing liver damage is unclear, but it could be explained by epigallocatechin gallate or its metabolite, epicatechin gallate, which, in certain conditions such as fasting, can induce oxidative stress and liver damage [269,270,271].

*Capsaicin* is the bioactive molecule of chili peppers that confers their pungent effects. In various pre-clinical models of liver disease, capsaicin exerted anti-inflammatory, anti-oxidant, anti-steatotic and anti-fibrotic effects through the stimulation of transient receptor potential vanilloid 1 channels. This activation, among other outcomes, triggers a cascade reaction that leads to an increase of PPAR system and GLP-1 activity [272]. Although no human studies are available assessing specifically the effects of capsaicin on liver diseases, its benefits on some components of the metabolic syndrome (chronic inflammation, insulin resistance, obesity, hypertension) make it an interesting molecule for future studies in patients with liver diseases. This interest is also fueled by its potential to increase satiety, resting metabolic rate and energy expenditure due to enhanced fat oxidation and gut microbiota modulation [273,274].

*Theobroma cacao* (cacao bean) is extremely rich in polyphenols (12–18% of its dry weight, comprising more than 200 molecules, with 60% being flavanols), acting as natural antioxidants by donating electrons to stabilize free radicals and by stimulating the transcription of various antioxidant enzymes [275]. Human consumption of dark chocolate derived from cacao beans reduces oxidative stress in patients with MAFLD [276], and pre-clinical studies have demonstrated a variety of beneficial effects on metabolic syndrome features, including arterial hypertension, inflammation, insulin resistance and steatotic liver [277,278].

#### 6.4.2. Prebiotics and Probiotics

An increasing interest is developing around the so-called gut–muscle axis, which correlates intestinal microbiota and muscle tissue mass and function [279]. Indeed, some animal studies have demonstrated that the administration of different probiotic strains is able to delay muscular age-related degeneration by altering the production of mediators, such as short-chain fatty acids (SCFA), cytokines and reactive oxygen species (ROS), thus restoring mitochondrial density and health. A recent randomized double-blind clinical trial in frail older adults parallelly demonstrated an improvement in muscle mass and function after the administration of Lactobacillus plantarum TWK10. This improvement was achieved by modulating intestinal microbiome and increasing muscular glycogen availability [280]. A few studies conducted on human cohorts have shown that prebiotic (inulin, oligosaccharides)-driven intestinal microbiological enrichment is associated with improved insulin sensitivity and frailty conditions, including grip strength [281,282]. Randomized clinical trials evaluating prebiotics (fructooligosaccharides, beta-glucan-supplemented cereals, psyllium husk, xylooligosaccharides, chicory inulin and fiber extracts), probiotics or synbiotics in the treatment of adult MASLD have been analyzed in several recent meta-analyses, very consistently reporting positive effects on liver enzymes. Despite this great amount of data, the strength of evidence is reduced by the heterogeneity of treatment combinations, their dosage and duration, as well as the limited availability of biopsy-supported MASLD/MASH diagnosis and histologic or imaging evaluation of treatment effects. Consequently, ESPEN guidelines on obesity care in patients with liver disease do not recommend the general use of pre/pro/symbiotics in these patients [205].

Dietary fiber administration protects from age-related sarcopenia by improving glucose metabolism, muscle function and lean body mass in adult subjects [283,284]. The underlying mechanism probably involves an increase in the production of short-chain fatty acids by gut microbiota, which are important regulators of skeletal muscle mass, metabolism and function [285], as well as an improvement in glucose homeostasis, with a decrease in insulin resistance and pro-inflammatory cytokine concentration [283,286].

## 7. Conclusions

The condition of sarcopenic obesity associated with liver cirrhosis of any origin is almost invariably characterized by a state of malnutrition, with an excess of caloric intake and a simultaneous deficit in proteins and micronutrients. Despite a clear association between sarcopenia/sarcopenic obesity and poor outcome in cirrhosis, nutritional interventions are based on pathophysiological assumptions, and only a few data are available on the impact of reversing this dysmetabolic condition on clinical endpoints in cirrhosis.

International nutrition and liver diseases societies strongly agree on recommending the use of food as an integral part of the healing process in the comprehensive management of these patients, including nutritional evaluation, personalized nutritional plan prescription and regular re-assessment of all cirrhotic patients. Nutritional intervention should aim at reducing weight and fat mass while preserving and, if possible, increasing lean mass and function, with an adequate supply of micronutrients. The best schedule to obtain these results consists in an overall daily energy intake of 25–35 kcal/kg and 1.2–1.5 g of proteins/kg (both calculated by referring to the ideal weight) split up in three main meals and three snacks, the last of them to be eaten right before bedtime in order to minimize the night-starvation period. The supplementation of nutrients should be considered in case these goals are not achieved with a personalized nutritional program, or in cases of proven deficiencies. In some particular cases, due to widely assessed pathophysiological rationales, the supplementation of some elements could be considered anyway; for example supplementation of BCAA and zinc in the case of hepatic encephalopathy or thiamine can be considered to prevent the Wernicke–Korsakoff syndrome in alcohol-related liver disease. In other situations, characterized by highly frequent deficiencies (decompensated patients, cholestatic liver disease or chronic alcohol abuse), where vitamin status is not easily assessed and multivitamin supplementation is cheap and free of substantial side effects, a course of oral multivitamin supplementation could be justified.

Other nutraceuticals, including omega-3 polyunsaturated fatty acids, L-carnitine, antioxidants, minerals such as magnesium and selenium and pre/probiotics, may display positive effects on energy metabolism, muscle homeostasis and liver function, but current evidence is largely observational and highly variable with regard to study designs and considered outcomes. For these reasons, further evidence to support their widespread use in these patients is required.

## 8. Case Study

Here we report a case study of a 60-year-old man with sarcopenic obesity and liver cirrhosis followed in our outpatient clinic. His anthropometrics are as follows: height 175 cm, weight 102 kg and BMI 33 kg/m^2^. The patient is independent in his movements but leads a sedentary lifestyle. He works as a public employee, spending more than 7 h daily in the office, mostly sitting.

According to his BMI, his ideal body weight is roughly 75 kg and, in accordance with the above-mentioned guidelines, his recommended daily energy intake is calculated as 30 kcal/kg of ideal body weight/day including a protein intake of 1.2 to 1.5 g/kg of ideal body weight. Applying these formulas to the patient’s ideal weight of 75 kg, we obtain:

- Daily energy intake of 2250 kcal, rounded to 2300 kcal;
- Protein intake between 90–113 g/day.

We developed a sample menu, based on the Mediterranean diet, organizing food intake into three main meals (breakfast, lunch, and dinner) and three snacks (midmorning, midafternoon, and late evening snack, LES). We included functional foods rich in proteins, omega-3 fatty acids, vitamins, and minerals. To achieve a medium of 30 gr of fiber, we proposed whole cereals, seasonal fruits, and vegetables. We also included Mediterranean herbs and spices to flavor dishes in order to limit the use of salt dressings.

CALORIC AND MACRONUTRIENT DISTRIBUTION:

- Energy: 2300 kcal (30 kcal/ideal body weight);
- Protein: 113 g (1.5 g/kg ideal weight);
- Carbohydrate: 240 g;
- Fat: 102 g;
- Saturated fat: 27 g (10% total kcal);
- Sodium: 1600 mg;
- Fiber 40 gr.

1. Breakfast: whole Greek yogurt with 60 g of oat flakes and blueberries (proteins 16 g);
2. Midmorning snack: a cube (30 g) of Grana Padano cheese and raw vegetables, such as a carrot and fennel (proteins 13 g);
3. Lunch: a single dish consisting of: 80 g of whole spelt with 100 g of cooked chickpeas, fresh cherry tomatoes, rocket, zucchini and one boiled egg. Dressing: three teaspoons of extra virgin olive oil, basil and chives as aromatic herbs (proteins 32 g);
4. Midafternoon snack: a whole apple with the peel and 30 g of sweet almonds (proteins: 7 g);
5. Dinner: 100 g of wild salmon, pan-cooked chicory, and a portion of baked potatoes. Dressing: three teaspoons of extra virgin olive oil, rosemary, garlic and chilly to flavor the dish (proteins: 27 g);
6. Late evening snack (LES): one slice of rye bread with two slices of cooked ham (proteins: 18 g, 250 kcal).

The patient was advised to drink at least 1.5 L of water a day, with hot drinks such coffee, green tea or herbal tea consumed without added sugar. The consumption of sugary and soft drinks was strongly discouraged.

## Data Availability

Not applicable.
